# Population attributable risks of modifiable reproductive factors for breast and ovarian cancers in Korea

**DOI:** 10.1186/s12885-015-2040-0

**Published:** 2016-01-06

**Authors:** Boyoung Park, Sohee Park, Hai-Rim Shin, Aesun Shin, Yohwan Yeo, Ji-Yeob Choi, Kyu-Won Jung, Byoung-Gie Kim, Yong-Man Kim, Dong-Young Noh, Sei-Hyun Ahn, Jae Weon Kim, Sokbom Kang, Jae Hoon Kim, Tae Jin Kim, Daehee Kang, Keun-Young Yoo, Sue K. Park

**Affiliations:** 1 Graduate School of Cancer Science and Policy, Goyang, Korea; 2 National Cancer Control Institute, National Cancer Center, Goyang, Korea; 3 Department of Epidemiology and Health Promotion, Graduate School of Public Health, Yonsei University, Seoul, Korea; 4 Western Pacific Regional Office, World Health Organization, Manila, Philippines; 5 Department of Preventive Medicine, Seoul National University College of Medicine, 103 Daehakro, Chongno-gu, Seoul 110-799 Korea; 6 Department of Biomedical Science, Seoul National University Graduate School, Seoul, Korea; 7 Department of Obstetrics and Gynecology, Samsung Medical Center & Sungkyunkwan University School of Medicine, Seoul, Korea; 8 Department of Obstetrics and Gynecology, University of Ulsan College of Medicine, Asan Medical Center, Seoul, Korea; 9 Department of Surgery and Cancer Research Institute, Seoul National University College of Medicine, Seoul, Korea; 10 Department of Surgery, University of Ulsan College of Medicine, Asan Medical Center, Seoul, Korea; 11 Department of Obstetrics and Gynecology, Seoul National University Hospital and Seoul National University College of Medicine, Seoul, Korea; 12 Department of Gynecologic Oncology, National Cancer Center, Goyang, Korea; 13 Department of Obstetrics and Gynecology, Gangnam Severance Hospital, Yonsei University College of Medicine, Seoul, Korea; 14 Department of Obstetrics and Gynecology, Cheil General Hospital and Women’s Healthcare Center, Dankook University Medical College, Seoul, Korea; 15 Cancer Research Institute, Seoul National University, Seoul, Korea

**Keywords:** Population attributable fraction, Breast cancer, Ovarian cancer, Modifiable factors, Reproductive factors

## Abstract

**Background:**

Breast and ovarian cancers are predominant female cancers with increasing prevalence. The purpose of this study was to estimate the population attributable risks (PARs) of breast and ovarian cancer occurrence based on the relative risks (RRs) of modifiable reproductive factors and population-specific exposure prevalence.

**Methods:**

The PAR was calculated by using the 1990 standardized prevalence rates, the 2010 national cancer incidence with a 20 year lag period, the meta-analyzed RRs from studies conducted in the Korean population for breast cancer, and the meta-analyzed RRs from a Korean epithelial ovarian cancer study and a prior meta-analysis, and ovarian cancer cohort results up to 2012. For oral contraceptive and hormone replacement therapy use, we did not consider lag period.

**Results:**

The summary PARs for modifiable reproductive factors were 16.7 % (95 % CI 15.8–17.6) for breast cancer (2404 cases) and 81.9 % (95 % CI 55.0–100.0) for ovarian cancer (1579 cases). The modifiable reproductive factors included pregnancy/age at first birth (8.0 %), total period of breastfeeding (3.1 %), oral contraceptive use (5.3 %), and hormone replacement therapy use (0.3 %) for breast cancer and included breastfeeding experience (2.9 %), pregnancy (1.2 %), tubal ligation (24.5 %), and oral contraceptive use (53.3 %) for ovarian cancer.

**Conclusions:**

Despite inherent uncertainties in the risk factors for breast and ovarian cancers, we suggest that appropriate long-term control of modifiable reproductive factors could reduce breast and ovarian cancer incidences and their related burdens by 16.7 % and 81.9 %, respectively.

**Electronic supplementary material:**

The online version of this article (doi:10.1186/s12885-015-2040-0) contains supplementary material, which is available to authorized users.

## Background

Worldwide, breast cancer is the most common cancer among females, and its incidence is increasing continuously. Ovarian cancer is the third most common gynecological cancer worldwide, next to cervix uteri and corpus uteri cancers, and has the second highest mortality rate among gynecological cancers, following that for cervix uteri cancer. Globally in 2008, breast and ovarian cancers accounted for 26.6 % of all cancers among females [1]. The main risk factors for breast cancer are reproductive factors such as age at menarche, number of births (parity), age at first birth, lactation (breastfeeding), and age at menopause [2]. Ovarian cancer is also influenced by reproductive risk factors such as parity, breastfeeding, and oral contraceptive (OC) use [3]. Each of these reproductive factors is associated with changes in circulating estrogen and progesterone levels and can be controlled by exogenous hormone treatment, such as OC use and menopausal hormone replacement therapy (HRT). Epidemiological studies regarding hormonal factors support the hypothesis that female hormones, particularly exogenous hormones such as those used for OC and HRT purposes, play an important role in the development of breast and ovarian cancers in women [4, 5].

In Korea, breast and ovarian cancers account for 14.3 % and 2.0 %, respectively, of all female cancers. In Korea, breast cancer is the second most common cancer following thyroid cancer, while ovarian cancer is ranked as 10^th^ most common among females [6]. Breast and ovarian cancer incidences have been continuously increasing over the past 20 years [7], and the average annual percentage increases in breast and ovarian cancers are 6.3 % and 1.6 %, respectively [6]. Increases in the prevalence of breast and ovarian cancers have been linked to rapid changes in reproductive factors, including age at menarche, menopause, parity, and birth-related characteristics (i.e., age at first birth, number of births, and breastfeeding), as well as a rapidly ageing population this country [8–10]. Particularly in Korea, rapid development and economic growth since the 1950 Korean War have given rise to marked westernization, leading to rapid changes in the reproductive risk factors of cancers. In 2005, Korea implemented a nationwide breast cancer screening program, which may be a contributor to the increased prevalence of breast cancer cases observed in this country.

The epidemiological patterns of breast and ovarian cancers and their risk factors in Korean women may require the development of population-specific strategies for cancer prevention. The determination of population attributable risks (PARs), defined as the quantified contribution of each risk factor to a disease, can help policymakers establish appropriate public health interventions [11].

The purpose of this study was to estimate the burden of reproductive risk factors on the prevalence of breast and ovarian cancer in Korea using Korean-specific risk estimates. Since breast and ovarian cancers are major female cancers in the Korean population, such population-specific prevalence and risk estimates should help in the development of cancer control plans in Korea.

## Methods

### Selection of major risk factors

Prior studies focused on breast cancer risk factors in Korea were based on a subset of data from the Seoul Breast Cancer Study (SeBCS) [12–17], the largest community based case–control study between 1993 and 2007. The cases consisted of women diagnosed with histologically confirmed breast cancer from three teaching hospitals located in Seoul, accounting for about 15-18 % of total breast cancer cases in Korea. The controls were composed of non-cancer patients or health examinees visiting the hospitals located in Seoul and near metropolitan areas. After getting written informed consent, information on demographic characteristics, reproductive factors, and lifestyle habits were collected by trained interviewers using a structured questionnaire. The SeBCS cases and controls were frequency matched by 5-year age and three enrollment period categories (1993–1997, 1998–2000, and 2001–2007). As a result, 3789 case and control sets were included in our analysis. Details of the SeBCS are described elsewhere [18].

In our previous studies using subset of data from the SeBCS, we have identified various risk factors of breast cancer in Korea including early menarche, late menopause, nulliparity, later first full-term pregnancy, family history, postmenopausal obesity, breastfeeding, and OC use [12–17]. For PAR calculations of potential risk factors, estimation of odds ratio (OR) for the full data set from the SeBCS, not a subset, was needed to get a small range for the 95 % confidence interval (CI). Next, we performed a pooled data analysis and selected variables according to the results from the multiple logistic regression. As a result, age at menarche, age at menopause, pregnancy age at first birth, total period of breastfeeding, and OC use were selected as significant reproductive factors. Of them, we chose pregnancy/age at first birth, total period of breastfeeding, and OC use as modifiable reproductive factors. “HRT use” was not a significant variable according to our results. However, we chose to include it since the IARC has reported that HRT is a carcinogenic agent in humans [19]. Although we selected risk factors for our new analysis, the selected risk factors of breast cancer were the same as those previously recognized as risk factors in Korea.

For ovarian cancer, Holschneider et al. reviewed the literature and proposed family history, genetic mutations, nulliparity, late menopause, and early menarche as risk factors for ovarian cancer. In addition, they suggested that multiparity, oral contraceptive use, and hysterectomy or tubal ligation were protective factors [20]. We selected reproductive factors that have previously been recognized as causal factors and the Korea Epithelial Ovarian Cancer Study (Ko-Eve) data was applied to estimate Korean OR values. The Ko-Eve study, started in 2009, is the only ongoing study in Korea. This community based case–control study covers incident epithelial ovarian cancers from six major centers and healthy controls among health examinees from community hospitals located in Seoul. Standardized questionnaires including socio-demographics characteristics, past medical history, family history, lifestyle habits, and reproductive factors for women were administered by trained interviewers. The details of the Ko-Eve are described elsewhere [21]. Initially, we analyzed the 231 cases registered from 2009 to 2011 and a group of 1:4 matched community controls (N = 924). We estimated Korean OR values using our ovarian cancer data from multiple logistic regression models (backward) adjusted for age, education, and alleged risk factors reported in the literature. In this process, family history of breast cancer, family history of ovarian cancer, age at menarche, age at menopause, pregnancy, breastfeeding, tubal ligation, and OC use were significant factors and were chosen. Afterwards, we selected pregnancy, breastfeeding, and tubal ligation, and OC use as modifiable factors.

Considering the effect of the population control policies through national birth control programs on the rapid decline of the fertility rate in Korea [22], we included ‘pregnancy/age at first birth’ for breast cancer and ‘pregnancy’ for ovarian cancer as modifiable factors. The variable pregnancy represented full-term pregnancy excluding miscarriages and induced abortion.

### Prevalence of exposure factors and cancer incidence

We applied the number of cancer incidents in the female population aged 20 years and older in the year 2010 from the Korea Central Cancer, a nationwide cancer registry in Korea [6]. Although several previous studies regarding PARs have suggested approximately a 20-year induction period, from exposure to risk to cancer development [23–26], studies regarding PARs of reproductive factors often suggested no lag time [27, 28]. In addition, for OC, the increased breast cancer risk disappears approximately 10 years after cessation of use [29], and cancer risk decreases rapidly after cessation of HRT use. Thus, no-lag time was considered and the prevalence in 2010 was estimated. We estimated the exposure prevalence of each selected modifiable reproductive risk factor in Korean females by using the data from the Korea National Health and Nutrition Examination Survey (KNHANES), which was performed on a random representative sample of the Korean population in 2005 [30]. Because the KNHANES did not include the history of tubal ligation, we determined the prevalence of tubal ligation based on the control subjects in the Ko-Eve study [21]. We estimated prevalence by applying an age-specific prevalence rate by 5-year age categories from the KNHANES 2005 or the Ko-EVE studies to the female populations in 2010 and summed up the totals to obtain standardized prevalence rates.

### Meta-analysis for estimation of risk for breast and ovarian cancers

To obtain the pooled relative risks (RRs) for the selected risk factors, we conducted a meta-analysis of the results of large-scale, case–control studies in Korea (SeBCS for breast cancer and Ko-Eve for ovarian cancer) and the results from other previous studies. For breast cancer analysis, because the SeBCS included large numbers of cases and matched controls, we restricted the data selection to studies conducted in Korea and did not restrict the study design to reflect Korean risk estimates. For ovarian cancer analysis, given that the Ko-Eve is the only study conducted in Korea, and includes a limited number of cases, we included data from international studies to perform a meta-analysis with Ko-EVE results to obtain stable risk estimates. The priority for inclusion of international data was meta-analysis or pooled analysis data. In cases where studies were not available, we included cohort study results. In cases where we could not obtain RR or the raw data necessary for calculating a RR estimate, the data were excluded from the meta-analysis.

Studies published in English or Korean before December 2012 were identified through PubMed (http://www.ncbi.nlm.nih.gov/pubmed/), Embase (http://www.embase.com/home), and KoreaMed (http://www.koreamed.org/SearchBasic.php). The search keywords were cancer (breast cancer, ovarian cancer) and each of the risk factors. For each article, we checked whether data resources overlapped. When more than one study from the same study population was available, the study with the most complete data was used.

For breast cancer, in addition to the SeBCS, one study [31] was included for meta-analysis of OC and HRT use, and only the results from the SeBCS were applied for pregnancy/age at first birth and duration of breastfeeding because there were no prior study results in Korea. For ovarian cancer, in addition to the Ko-Eve, 11 cohort studies [32–42] were identified for pregnancy, but two studies [35, 38, 39] were excluded because of shared study populations ([39, 40] and [35, 41]) and one study of women with infertility problems [38] was excluded. In order to obtain pooled RRs, four cohort studies [36, 39, 42, 43] were included for breastfeeding analysis (Additional file 1: Figure S1) and meta-analysis studies were included for each tubal ligation [44] and OC use [45], in addition to the Ko-Eve. Meta-analysis estimates from both cohort studies and case–control studies were applied to achieve our combined results. In the meta-analysis, we did not access the inconsistency, publication bias, and other risk of bias. Stata version 12.0 (StataCorp, College Station, TX, USA) was used for the meta-analysis. The results from the included or excluded studies are summarized in Additional file 2: Table S1. The study design and the present study were approved by the Seoul National University institutional review board in compliance with the Helsinki Declaration (IRB number: C-0909-048-295).

### Statistical analysis

From the estimated prevalence of exposure in the population (*p*) and the RRs for each particular risk factor, the PAR for each risk can be calculated. The PAR was calculated by using the modified Levin’s formula for multiple categories, as proposed by Hanley [46, 47].

$$ \mathrm{P}\mathrm{A}\mathrm{F}={\displaystyle \sum {p}_i}\left({\mathrm{RR}}_i-1\right)/\left(1+{\displaystyle \sum {p}_i\left({\mathrm{RR}}_i-1\right)}\right) $$

PARs of breastfeeding duration for breast cancer, as well as breastfeeding experience and tubal ligation for ovarian cancer were estimated using the prevalence of pregnancy (97 %). PARs of HRT use for breast cancer was estimated using the prevalence of menopause (32 %) in women aged 20 years or over in 2010.

By using the obtained PARs, we calculated the proportions and numbers of cases and deaths of breast and ovarian cancer due to modifiable reproductive factors in females 20 years of age and older.

## Results

The RRs and prevalences for breast and ovarian cancer applied in the current study along with the data sources are summarized in Table 1. A later first pregnancy age showed a higher risk for breast cancer, and those whose total period of breastfeeding was ≤6 months showed an increased breast cancer risk (RR = 1.28 [95 % CI 1.07–1.53]) compared with females with a breastfeeding period of ≥7 months. The use of OC was associated with a 1.31-fold higher risk for breast cancer in the pooled analysis (95 % CI 1.04–1.64); however, HRT use was not significantly associated with breast cancer (RR = 1.16 [95 % CI 0.36–3.78]). Nulliparous women had a 1.42-fold higher risk for ovarian cancer (95 % CI 1.31–1.54). Women who did not have breastfeeding experience had an increased risk of ovarian cancer (RR = 1.17 [95 % CI 1.02–1.33]). Female who did not undergo tubal ligation had an increased risk of ovarian cancer (RR = 1.44 [95 % CI 1.33–1.56]) and those who did not have experience to take OC also had an increased risk (RR = 1.87 [95 % CI 0.89–3.94]).

**Table 1:** Summary of relative risks and prevalence (%) of exposure to modifiable reproductive factors in Korean women and data source

Risk factors	Category	Relative risk (95 % CI)	Study design	Source	Prevalence (%)^a^	Source
Breast cancer						
Pregnancy/age at first birth	Nulliparous	1.06 (0.82-1.36)	Case–control study	SeBCS	3	KNHANES, 2005
	≤23 years	1.00			36	
	24 – 30 year	1.13 (0.98-1.32)			57	
	≥31 year	1.27 (0.98-1.66)			4	
Total period of breast feeding	Never	1.03 (0.87-1.21)	Case–control study	SeBCS	17 (18^b^)	KNHANES, 2005
	≤6 months	1.28 (1.07-1.53)			9 (10^b^)	
	≥7 months	1.00			70 (72^b^)	
Oral contraceptive use	Never	1.00	Meta-anlaysis	SeBCS and [31]	82	KNHANES, 2005
	Ever	1.31 (1.04-1.64)			18	
Hormone replacement therapy use	Never	1.00			30 (95^c^)	KNHANES, 2005
	Ever	1.16 (0.36-3.78)	Meta-anlaysis	SeBCS and [31]	2 (5^c^)	
Ovarian cancer						
Pregnancy	Nulliparous	1.42 (1.31-1.54)	Meta-anlaysis	Ko-EVE, [32–34, 36, 37, 40–42]	3	KNHANES, 2005
	Parous	1.00			97	
Breast feeding experience	Never	1.17 (1.02-1.33)	Meta-anlaysis	Ko-EVE, [36, 39, 42, 43]	17 (18^b^)	KNHANES, 2005
	Ever	1.00			79(82^b^)	
Tubal ligation	No	1.44 (1.33-1.56)	Meta-anlaysis	Ko-EVE and [44]	75 (77^b^)	Ko-EVE^d^, 2009-2011
	Yes	1.00			22 (23^b^)	
Oral contraceptive use	Never	1.87 (0.89-3.94)	Meta-anlaysis	Ko-EVE and [45]	82	KNHANES, 2005
	Ever	1.00			18	

^a^Standardized prevalence rates among women aged 20 or over by 1990 Korean population census data for pregnancy/age at first birth, total period of breast feeding, pregnancy, breast feeding experience, and tubal ligation. Standardized prevalence rates among women aged 20 or over by 2010 Korean population census data for oral contraceptive use and hormone replacement therapy

^b^Prevalence in parous women. In the Korean population, the prevalence of women with experience in breastfeeding was 97 %

^c^Prevalence in post-menopausal women. In the Korean population, the prevalence of post-menopausal women was 32 %

^d^Used control population only

The PARs and numbers of breast and ovarian cancer incidences due to modifiable reproductive factors among females aged 20 years and older for the year 2010 in Korea are presented in Table 2. Pregnancy/age at first birth was the most important modifiable reproductive factor for breast cancer (PAR = 8.0 %), followed by OC use, total period of breastfeeding, and HRT use, which were attributed to 5.3 %, 3.1 %, and 0.3 %, respectively, of breast cancer incidences. The PAR for the selected modifiable reproductive factors was 16.7 % (95 % CI 15.8–17.6) for breast cancer, and those factors were responsible for 2,404 (95 % CI 2,283–2,535) breast cancer cases in the year of 2010.

**Table 2:** The population attributable risks and estimated number of new cancer cases in Korean women caused by modifiable reproductive factors in the year 2010

Cancer site	Risk factors	Population attributable risks % (95 % CI)	Attributed N of cases^c^
Breast	Pregnancy/age at first birth	8.0 (7.4-8.6)	1152 (1066–1246)
	(Nullipara or age at first birth ≥ 24 years)		
	Total period of breast feeding^a^	3.1 (3.1-3.2)	453 (441–464)
	(Duration of breast feeding ≤ 6 months)		
	Oral contraceptive use (Ever)	5.3 (5.1-5.5)	763 (732–795)
	Hormone replacement therapy use (Ever)^b^	0.3 (0.2-0.3)	37 (29–44)
	Total	16.7 (15.8-17.6)	2404 (2283–2535)
Ovary	Pregnancy (Nulliparous)	1.2 (1.2-1.2)	24 (24–24)
	Breast feeding experience (Never)^a^	2.9 (2.8-2.9)	55 (55–56)
	Tubal ligation (No)^a^	24.5 (23.0-26.2)	473 (444–504)
	Oral contraceptive use (Never)	53.3 (27.9-100.0^d^)	1027 (537–1927)
	Total	81.9 (55.0-100.0 ^d^)	1579 (1059–1927)
			Attributed % of cases
	% of breast cancer in women		16.7 (15.8-17.6)
	% of ovary cancer in women		81.9 (55.0-100.0^d^)

^a^PAFs were considered only for parous females (97 % of total females)

^b^PAFs were considered only for post-menopausal females (32 % for total females)

^c^Attributable number of cases = population attributable risk X numbers of breast or ovarian cancer incidence in the year 2010 from National Cancer Registry

^d^The population attributable risk exceed 100 (102.1) and truncated to 100

For ovarian cancer, OC use and tubal ligation were the most important modifiable reproductive factors (PAR = 53.3 % [95 % CI 27.9-100.0] and PAR = 24.5 % [95 % CI 23.0–26.2], respectively), whereas breast feeding and pregnancy were attributed to 2.9 % and 1.2 %, respectively. The PAR for the selected modifiable reproductive factors was 81.9 % (95 % CI 55.0–100.0) of ovarian cancer cases and they were responsible for 1579 (95 % CI 1059–1927) ovarian cancer incidences in the year of 2010 in Korea.

## Discussion

In Korea in the year 2010, 16.7 % of breast cancer and 81.9 % of ovarian cancer cases in women were attributable to modifiable reproductive factors. The modifiable reproductive factors included pregnancy/age at first birth (8.0 %), total period of breastfeeding (3.1 %), OC use (5.3 %), and HRT use (0.3 %) for breast cancer and included pregnancy (1.2 %), breastfeeding (2.9 %), tubal ligation (24.5 %), and OC never use (53.3 %) for ovarian cancer.

Several recent studies have reported PARs of reproductive factors for breast cancer. In one study, a combination of parity number and age at first birth explained 17.9 % of breast cancers [48], a percentage higher than that in our results for age at first birth only (8.4 %). However, that study’s overall PAR of breast cancer attributable to reproductive factors was similar to ours. Barnes et al. included the most reported risk factors for breast cancer including modifiable and non-modifiable factors in their PAR calculation and showed that about 50 % of breast cancers in post-menopausal women were attributed to hormone and reproductive factors such as age at menarche (7.7 %), age at menopause (12.0 %), parity (10.9 %), and HRT use (19.4 %) [49]. Parkin et al. considered only breastfeeding and attributed it to 3.2 % of female breast cancers [50]. A study from China showed that 6.7 % of breast cancers in women aged 15–49 years were attributed to reproductive factors, which included parity, number of children, age at first birth, and breastfeeding, and 0.7 % and 0.3 % were attributed to OC use and HRT use, respectively [28].

Among the assessed modifiable reproductive factors, large differences in PARs between studies were observed in HRT use. In French women, HRT use was associated with 12.7 % of breast cancer cases and 10.2 % of breast cancer deaths [27], whereas HRT use was attributed to 19.4 % [49], 3.2 % [50], 0.3 % [28], 4.4 % [51], and 2.4 % [52] of female breast cancer in Germany, the United Kingdom, China, the United States, and Japan, respectively. In the Korean population, the PAR of HRT was very low (0.3 %), similar to that reported for China [28]. The low level in Korea may be because HRT use is not common in Korea (5 % in post-menopausal women) and its RR is low.

Regarding the classification of risk factors, parity was considered a non-modifiable factor in the work of Barnes et al., but was assessed as a modifiable factor in this study. Barnes et al. restricted their PAR calculation to post-menopausal women, and parity-related factors could thus be considered as non-modifiable [49]. In contrast, the present study included all women aged 20 or older, and parity, pregnancy/age at first birth, and total period of breastfeeding were thus classified as modifiable factors. The International Agency for Research on Cancer and France working group estimated PAR changes from 1980 to 2000 by assessing changes in reproductive factors including parity (nulliparous vs. parous), mean number of children, age at first birth, and breastfeeding duration, and showed that changes in reproductive factors over those 20 years were associated with 6.7 % and 0.38 % of breast and ovarian cancers increases, respectively [27]. Those results indicate that such factors can produce temporal changes in cancer incidence. Korea has an experience in decreasing fertility rate fast (from 6.0 births per woman in 1960 to 1.08 in 2005 and to 1.23 in 2010) through national birth control program as part of a population control policies began in 1958. Although the decreasing fertility rate is common phenomenon worldwide, the speed of decrement is one of the fast and now fertility rate in Korea is the lowest in the world [22]. So, Korean government has changed family planning policy to childbirth encouragement and tries to increase the birth rate intensively with many benefits to families. Considering that the PAR can help policy makers establish appropriate public health interventions and efforts to control the birth rate in Korea for about past 60 years, including pregnancy/age at first birth as modifiable factors would helpful for policymakers not only in Korea but also other countries which have family planning policy to support their recommendation about childbirth. Thus, under this new policy, the encouragement of childbirth, particularly at an early age, might reduce breast and ovarian cancer incidences and deaths.

With regard to the PAR in ovarian cancer, Granstrom et al. reported that the PARs for family history, parity/age at first birth, and residential area were 2.6 %, 22.3 %, and 7.2 %, respectively [53]. Parazzini et al. included more risk factors, and the PARs were 4 % for a family history of breast and ovarian cancer, 8 % for age at menopause, 5 % for parity, 12 % for OC use, 7 % for high fat intake score, and 24 % for low green vegetable intake [54]. Parkin et al. considered only HRT use as a reproductive risk factor for ovarian cancer and attributed 0.7 % of the ovarian cancer incidence to HRT. The OC use is a protective factor for ovarian cancer [55] and in Korea the PAR of OC was higher than other studies because prevalence OC use was very low (18 %). As a contraceptive method, tubal ligation was associated with a lower risk of ovarian cancer and, during the era of encouraging birth control in Korea, the most common artificial sterilization method was tubal ligation. Therefore, the PAR of tubal ligation for ovarian cancer was also higher.

There are several study limitations to be considered. Although the standardized population used in this study was from 1990, prevalence of most reproductive factors in 2005 was used because of the lack of representative data for 1990. Considering the increased age at first birth and the decreased fertility rate between 1990 and 2005, our results might underestimate the PAR for reproductive factors in Korea. In addition, the prevalence of tubal ligation showed limited representativeness because it was estimated by using control subjects from a hospital-based case–control study (Ko-Eve). Thus, we compared our estimates of the prevalence of tubal ligation with results from the National Survey on Fertility, Family Health & Welfare in Korea, a nationwide representative survey for females of childbearing age (15–44 years) [56], and the results were comparable to ours (18.3 % vs. 23 %, respectively). Moreover, our estimates of RRs were based on a limited number of studies, which may have introduced uncertainty in the pooled RR estimate and, hence, uncertainty in the calculated PARs. Although several review articles and meta-analysis studies have reported stable results for RR estimates of breast and ovarian cancer risk factors, in this study, we used Korean-specific results. We considered ethnicity- or country-specific risk estimates, distributions, and their effects on PAF estimates. In addition, while we did not consider the quality of each study included in our RR estimation, Korean studies included in the breast/ovarian cancer RR estimation were community based case–control studies. For ovarian cancer, we pooled results from previous international studies with those from a Ko-Eve. Therefore, the RR values might be less appropriate for the Korean female population. However, due to the lack of Korean data and followed by instable results for ovarian cancer, pooled estimates with previous international studies would be unavoidable as Shin et al. did in the previous study [26]. The cut-off points were arbitrary and identified for convenience in our meta-analysis.

Despite these limitations, this study has several strengths. First, we used nationwide cancer incidence data that are representative of nearly the entire population. Thus, we had access to precise numbers of cancer cases for inclusion in our PAR estimation. Second, the estimated prevalence of exposure to each risk factor in 1990 was used in the consideration of a 20-year lag period between exposure to a risk factor and subsequent cancer development. Although we did not measure the quality of each study, the studies included in the meta-analysis for breast cancer were conducted within the Korean population, thus providing Korean-specific results.

## Conclusions

In summary, the results of this study represent a systematic assessment of breast and ovarian cancer risks and the proportion of the risk associated with modifiable reproductive factors. A total of 16.7 % of breast cancer cases (2404 cases) and 81.9 % of ovarian cancer cases (1579 cases) in Korea among female individuals 20 years of age in 2010 were attributable to modifiable reproductive factors. Since breast and ovarian cancers are the most prevalent female cancers, and are showing a trend to higher prevalence, appropriate control of preventable or modifiable risk factors is an important strategy for reduction of the female cancer burden in Korea. Combining the current Korean family planning policy of childbirth encouragement with cancer control strategies that affect modifiable reproductive factors may help achieve reductions in breast and ovarian cancer incidences.

## Additional files

Additional file 1: Figure S1. Flow chart of the study selection for breast and ovarian cancer risk estimates. (DOCX 658 kb) Additional file 2: Table S1. Studies included and excluded in the pooled-analysis of risk factors to calculate population attributable risks in breast and ovarian cancers. (DOC 138 kb)

## Abbreviations

CI: confidence interval
HRT: hormone replacement therapy
KNHANES: Korea National Health Examination Survey
Ko-Eve: Korea Epithelial Ovarian Cancer Study
OC: oral contraceptive
OR: odds ratio
PAR: population attributable risk
RR: relative risk
SeBCS: Seoul Breast Cancer Study
