# Erratum to: Population attributable risks of modifiable reproductive factors for breast and ovarian cancers in Korea

**DOI:** 10.1186/s12885-016-2216-2

**Published:** 2016-03-04

**Authors:** Boyoung Park, Sohee Park, Hai-Rim Shin, Aesun Shin, Yohwan Yeo, Ji-Yeob Choi, Kyu-Won Jung, Byoung-Gie Kim, Yong-Man Kim, Dong-Young Noh, Sei-Hyun Ahn, Jae Weon Kim, Sokbom Kang, Jae Hoon Kim, Tae Jin Kim, Daehee Kang, Keun-Young Yoo, Sue K. Park

**Affiliations:** Graduate School of Cancer Science and Policy, Goyang, Korea; National Cancer Control Institute, National Cancer Center, Goyang, Korea; Department of Epidemiology and Health Promotion, Graduate School of Public Health, Yonsei University, Seoul, Korea; Western Pacific Regional Office, World Health Organization, Manila, Philippines; Department of Preventive Medicine, Seoul National University College of Medicine, 103 Daehakro, Chongno-gu, Seoul, 110-799 Korea; Department of Biomedical Science, Seoul National University Graduate School, Seoul, Korea; Department of Obstetrics and Gynecology, Samsung Medical Center & Sungkyunkwan University School of Medicine, Seoul, Korea; Department of Obstetrics and Gynecology, University of Ulsan College of Medicine, Asan Medical Center, Seoul, Korea; Department of Surgery and Cancer Research Institute, Seoul National University College of Medicine, Seoul, Korea; Department of Surgery, University of Ulsan College of Medicine, Asan Medical Center, Seoul, Korea; Department of Obstetrics and Gynecology, Seoul National University Hospital and Seoul National University College of Medicine, Seoul, Korea; Department of Gynecologic Oncology, National Cancer Center, Goyang, Korea; Department of Obstetrics and Gynecology, Gangnam Severance Hospital, Yonsei University College of Medicine, Seoul, Korea; Department of Obstetrics and Gynecology, Cheil General Hospital and Women’s Healthcare Center, Dankook University Medical College, Seoul, Korea; Cancer Research Institute, Seoul National University, Seoul, Korea

## Erratum

After publication of the original article [1], the authors noticed an error in the Acknowledgements section. One of the supporting grants is missing. The correct version of the Acknowledgements section is included below:
