# Supplementary material for: Population attributable risks of modifiable reproductive factors for breast and ovarian cancers in Korea
Source: BMC Cancer. 2016 Jan 6;16:5. doi: 10.1186/s12885-015-2040-0 (PMC4702325; doi:10.1186/s12885-015-2040-0)
Supplement: Additional file 1: Figure S1. — Flow chart of the study selection for breast and ovarian cancer risk estimates. (DOCX 658 kb) [file 12885_2015_2040_MOESM1_ESM.docx]

Additional file 1: Figure S1

(a) Breast cancer, pregnancy/age at first birth

Search term: (pregnancy) AND (age at first birth) AND (breast cancer) AND (Korea)

Filter:- Publication date: 01.01.1980-12.31.2012

- Language: English and Korean

(b) Breast cancer, period of breast feeding

Search term: (period of breast feeding) AND (breast cancer) AND (Korea)

Filter:- Publication date: 01.01.1980-12.31.2012

- Language: English and Korean

(c) Breast cancer, oral contraceptives

Search term: (oral contraceptives) AND (breast cancer) AND (Korea)

Filter:- Publication date: 01.01.1980-12.31.2012

- Language: English and Korean

(d) Breast cancer, hormone replacement therapy

Search term: (hormone replacement therapy) AND (breast cancer) AND (Korea)

Filter:- Publication date: 01.01.1980-12.31.2012

- Language: English and Korean

(e) Ovarian cancer, pregnancy

Search term: (pregnancy) AND (ovarian cancer) AND (prospective cohort study)

Filter:- Publication date: 01.01.1980-12.31.2012

- Language: English and Korean

(f) Ovarian cancer, breast feeding

Search term: (breast feeding) AND (ovarian cancer) AND (prospective cohort study)

Filter:- Publication date: 01.01.1980-12.31.2012

- Language: English and Korean

(g) Ovarian cancer, tubal ligation

Search term: (tubal ligation) AND (ovarian cancer) AND (meta-analysis)

Filter:-Article type: Meta analysis and Systematic review

- Publication date: 01.01.1980-12.31.2012

- Language: English and Korean

(h) Ovarian cancer, oral contraceptives

Search term: (oral contraceptives) AND (ovarian cancer) AND (meta-analysis)

Filter:-Article type: Meta analysis and Systematic review

- Publication date: 01.01.1980-12.31.2012

- Language: English and Korean
