# Supplementary material for: Population attributable risks of modifiable reproductive factors for breast and ovarian cancers in Korea
Source: BMC Cancer. 2016 Jan 6;16:5. doi: 10.1186/s12885-015-2040-0 (PMC4702325; doi:10.1186/s12885-015-2040-0)
Supplement: Additional file 2: Table S1. — Studies included and excluded in the pooled-analysis of risk factors to calculate population attributable risks in breast and ovarian cancers. (DOC 138 kb) [file 12885_2015_2040_MOESM2_ESM.doc]

**Additional file 2: table S1. Studies included and excluded in the pooled-analysis of risk factors to calculate population attributable risks in breast and ovarian** cancers

| Resources | Study design | Definition | No. of participants | Results |
| --- | --- | --- | --- | --- |
| **Breast cancer** |  |  |  |  |
| Seoul Breast Cancer Study | Community-based case-control study  (Prototype of SeBCS: Hospital-based case-control study) | Cases: newly diagnosed histologically breast cancer cases from 1993 to 2007  Controls: breast cancer-free population lived in the community from 1993 to 2007 | 3,789 1:1 matched sets by a 5-year age category and by enrollment year | < Pregnancy/age at first birth>   | Nulliparous | 1.06 (0.82-1.36) | | --- | --- | | ≤23 yrs | 1.00 | | 24 – 30 yrs | 1.13 (0.98-1.32) | | ≥31 yrs | 1.27 (0.98-1.66) |   < Total period of breast feeding>   | Never | 1.03 (0.87-1.21) | | --- | --- | | ≤ 6 months | 1.28 (1.07-1.53 | | ≥7 months | 1.00 |   < Oral contraceptive use>   | Never | 1.00 | | --- | --- | | Ever | 1.37 (1.1 |   -1.71  < Hormone replacement therapy use>   | Never | 1.00 | | --- | --- | | Ever | 0.69 (0.46-1.05) | |
|
|
|
|
|
|
|
|
|
|
|
| Lee et al., 2007 | Community-based case-control study | Cases: breast cancer cases from 2004 to 2005  Controls: breast cancer-free population lived in the community from 2004 to 2005 | 115 cases and 173 controls | < Oral contraceptive use>   | Never | 1.00 | | --- | --- | | Ever | 0.98 (0.54-1.78) |   < Hormone replacement therapy use>   | Never | 1.00 | | --- | --- | | Ever | 2.33 (0.86-6.35) | |
| **Ovarian cancer** |  |  |  |  |
| Korea Epithelial Ovarian Cancer Study | Community-based case-control study and ovarian cancer cohort study | Cases: newly diagnosed histologically epithelial ovarian cancer cases  Controls: ovarian cancer-free population lived in the community | 234 1:4 matched sets by a 5-year age category  (234 cases and 924 controls) | < Pregnancy >   | Nulliparous | 1.00 | | --- | --- | | Parous | 0.39 (0.15-0.95) |   < Breast feeding >   | Never | 1.00 | | --- | --- | | Ever | 0.53 (0.29-0.95) |   < tubal ligation>   | No | 5.70 (2.23-14.57) | | --- | --- | | Yes | 1.00 |   < Oral contraceptive use>   | Never | 2.98 (1.49-5.93) | | --- | --- | | Ever | 1.00 | |
| Hankinson et al., 1995 | Cohort study | Cohort: Nurses' Health Study participants  Outcomes: questionnaire and liked to the National Death Index | 121,700 women (260 epithelial ovarian cancer) | < Pregnancy >   | Nulliparous | 1.00 | | --- | --- | | Parous | 0.54 (0.37-0.80) | |
| Albrektsen et al., 1996 | Cohort study | Cohort: all Norwegian women born from 1935 through 1971 included in the Central Population Register  Outcomes: linked to the Cancer Registry of Norway and data on emigrations deaths from Central Bureau of Statistics | 1,145,076 women (1,694 epithelial ovarian cancer) | < Pregnancy >   | Nulliparous | 1.32 (1.13-1.53) | | --- | --- | | Parous | 1.00 | |
| Mink et al., 1996 | Cohort study | Cohort: 98,029 women 55–69 years of age randomly selected from the state driver’s license list in January 1986 (Iowa women's health study)  Outcomes: questionnaire and linked to the Iowa death certificates and the State Health Registry of Iowa, part of the Surveillance, Epidemiology, and End Results (SEER) Program | 31,396 postmenopausal women  (97 epithelial ovarian cancer) | < Breast feeding >   | Never | 1.00 | | --- | --- | | Ever | 1.03 (0.66-1.61) |   < Pregnancy >   | Nulliparous | 1.00 | | --- | --- | | Parous | 0.53 (0.30-0.91) |   Excluded for pregnancy: Shared population with Vachon et al. 2002 |
| Vachon et al., 2002 | Cohort study | Cohort: 98,029 women 55–69 years of age randomly selected from the state driver’s license list in January 1986 (Iowa women's health study)  Outcomes: questionnaire and linked to the Iowa death certificates and the State Health Registry of Iowa, part of the Surveillance, Epidemiology, and End Results (SEER) Program | 31,377 postmenopausal women aged 55-69  (181 epithelial ovarian cancer) | < Pregnancy >   | Nulliparous | 1.62 (1.04-2.52) | | --- | --- | | Parous | 1.00 | |
| Kumle et al., 2004 | Cohort study | Cohort: Randomly selected Norwegian women aged 30-49 from Central Population Register in Norway and the Swedish Central Population Registry at Statistics Sweden  Outcomes: linked to the national cancer registry | 103,551 women (214 epithelial ovarian cancer) | < Pregnancy >   | Nulliparous | 1.00 | | --- | --- | | Parous | 0.6 (0.4-0.9) | |
| Lacey et al., 2006 | Cohort study | Cohort: NIH-AARP Diet and Health Study participants: members between 50 and 71 years of age who resided in one of six US states  Outcomes: linkage to eight state cancer registries | 97,638 women (214 epithelial ovarian cancer) | Excluded study: Shared population with Yang et al. 2012 |
| Tsilidis et al., 2006 | Cohort study | Cohort: European Prospective Investigation into Cancer and Nutrition (EPIC) participants  Outcomes: linkage to population cancer registries, using a combination of methods including linkage to health insurance records, cancer and pathology registries, and active follow-up of study participants | 327,396 women (878 ovarian cancer) | < Pregnancy >   | Nulliparous | 1.00 | | --- | --- | | Parous | 0.71 (0.59-0.87) |   < Breast feeding >   | Never | 1.00 | | --- | --- | | Ever | 0.86 (0.70-1.07) | |
| Jensen et al., 2009 | Cohort study | Cohort: women with infertility referred to all Danish hospitals  or private infertility clinics during 1963-98  Outcomes: linkage to the Danish Cancer  Registry and the Danish Registry of Pathology | 54,362 women (156 epithelial ovarian cancer) | Excluded study: study population was not general female population but women with infertility problems |
| Braem et al., 2010 | Nested case-control study in the cohort | Cohort: the Netherlands Cohort Study on Diet and Cancer participants aged 55-69.  Outcomes: record linkage of the entire cohort to the Netherlands Cancer  Registry and the Netherlands Pathology Registry | 375 cases and 2,331 subcohort members(post-menopausal women) | < Pregnancy >   | Nulliparous | 1.00 | | --- | --- | | Parous | 0.68 (0.53-0.89) | |
| Yang et al., 2012 | Cohort study | Cohort: NIH-AARP Diet and Health Study participants: members between 50 and 71 years of age who resided in six states (California,  Florida, Louisiana, New Jersey, North Carolina, and Pennsylvania) and two metropolitan  areas (Atlanta, Georgia and Detroit, Michigan)  Outcomes: probabilistic linkages with cancer registries in the original recruitment areas | 169,391 women (849 epithelial ovarian cancer) | < Pregnancy >   | Nulliparous | 1.00 | | --- | --- | | Parous | 0.71 (0.61-0.85) | |
| Weiderpass et al. 2012 | Cohort study | Cohort: Japan Public Health Center-based Prospective Study,  which began in 1990 for cohort I, and in 1993 for cohort II,  included Japanese inhabitants living in the municipalities  served by 11 different public health centers. Participants were aged 40-59 years in  cohort I, and 40-69 years in cohort II at the time of enrollment  Outcomes: Linkage with data sources form major local hospitals and population-based cancer registries. Death certificates were used to supplement the information | 45,748 women (86 epithelial ovarian cancer) | < Pregnancy >   | Nulliparous | 1.3 (0.6-3.1) | | --- | --- | | Parous | 1.00 | |
| Danforth et al., 2007 | Cohort study | Cohort: Nurses’ Health Study and Nurses’ Health Study II  cohorts : registered nurses in 11 US states who completed  a self-administered, mailed questionnaire  Outcomes: responses  to biennial questionnaires or death certificates | 142,360 women (391 epithelial ovarian cancer) | < Breast feeding >   | Never | 1.00 | | --- | --- | | Ever | 0.86 (0.70-1.06) | |
| Rice et al., 2012 | Meta-analysis | Outcomes: association between ovarian cancer and tubal ligation | 30 studies (1984-2012) | < Tubal ligation >   | No | 1.00 | | --- | --- | | Yes^*^ | 0.70 (0.64-0.75) |   ^*^ Estimated from all of the selected studies (both case-control and cohort studues) |
| Beral et al., 2008 | Meta-analysis | Outcomes: association between ovarian cancer and oral contraceptives | 45 studies (up to January 2006) | < Tubal ligation >   | No | 1.00 | | --- | --- | | Yes^*^ | 0.73 (standard error: 0.02) |   ^*^ Estimated from all of the selected studies (both 32 case-control studies and 13 cohort studies) |
